# Are Conventional Thermochemical Calculations a Viable Alternative to Measurements of Vaporization Enthalpy of Azeotropes?

**DOI:** 10.3390/molecules30040810

**Published:** 2025-02-10

**Authors:** Eliza Kołodziejczyk, Wojciech Marczak

**Affiliations:** Faculty of Science and Technology, Jan Dlugosz University, Al. Armii Krajowej 13/15, 42-200 Czestochowa, Poland; eliza.kolodziejczyk@op.pl

**Keywords:** phase transition, Hess cycle, azeotrope, heat of vaporization, binary mixtures, thermodynamic cycle

## Abstract

The majority of the studies of vaporization enthalpy dealt with pure substances. Reports of this quantity for azeotropes were scarce despite that similar experimental methods could be applied in such measurements. Vaporization enthalpies of azeotropes were determined using classical methods in the past and with a method based on the enthalpy of solution recently. Since the reported results showed discrepancies that often exceeded the declared uncertainty limits, we calculated the vaporization enthalpies of 12 azeotropes at normal boiling temperature and 298.15 K using the conventional thermochemical cycle at several levels of approximation. We validated our calculation procedure and assessed the uncertainty of the results. The assessments were based on (*i*) a comparison of the calculated vaporization enthalpies with the experimental ones reported in the literature, and (*ii*) a Monte Carlo simulation involving 10^6^ trials with the independent variables characterized by continuous uniform distributions. The calculated vaporization enthalpies of the azeotropes proved to be correct even if they were only roughly approximated by the mole-fraction-weighted averages of the vaporization enthalpies of pure components. Thermochemical cycle calculations provided results at least as good as the experimental vaporization enthalpies, those obtained from the enthalpies of solution in particular.

## 1. Introduction

Vaporization enthalpy can be determined experimentally using many methods, from which the following five have been commonly applied in practice: static, ebulliometric, effusion, transpiration, and calorimetric [1]. The last one is the only method where the heat effect of the phase change is measured directly. The others consider rather the relationship between vapor pressure, *p*, and temperature, *T*, which is given by the Clausius–Clapeyron equation:

(1) $${\left(\frac{\partial p}{\partial T}\right)}_{G}=\frac{{∆}_{vap}H}{T{∆}_{vap}V}$$

where Δ_vap_*H* and Δ_vap_*V* are the enthalpy and volume of vaporization, and subscript *G* denotes the constant Gibbs energy of the system (e.g., [2]). Equation (1) is a thermodynamic strict relationship derived from the liquid–vapor equilibrium condition. To make the Δ_vap_*H* determination possible, various equations for the dependencies of the enthalpy and volume of vaporization on pressure and temperature have been introduced [3] because a universal equation of state for fluids has not been formulated yet.

The methods applying empirical relationships between vaporization characteristics and other measurable physicochemical quantities constitute the second group. For example, the retention volume [4] or the retention time [5] were measured in chromatographic experiments. The correlation gas chromatography method uses the linear correlation between enthalpies of transfer from the solution to the vapor, as measured by gas chromatography, and the vaporization enthalpy of a series of standards [5].

The solution calorimetry method stands apart from those enumerated here as it is based solely on the characteristics of the liquid phase, applying the experimental enthalpies of the solution rather than that of vaporization itself and uses two empirical relationships in the calculation procedure, e.g., [6]. The vaporization enthalpy of a substance A, Δ_vap_*H*_A_, was related to its solution enthalpy Δ_soln_*H*_A/S_ in an inert solvent (S) by the following formula:

(2) $${∆}_{vap}H_{A}={∆}_{soln}H_{A/S}-{∆}_{solv}H_{A/S},$$

where Δ_solv_*H*_A/S_ was the solvation enthalpy of the solute A in the solvent S. The solvation enthalpy Δ_solv_*H*_A/S_ was calculated from its relationship with the molar refraction of *R*_A_ of the substance A:

(3) $${-∆}_{solv}H_{A/S}=a+b\cdot R_{A},$$

where *a* and *b* were empirical coefficients dependent on the solvent [6].

The vaporization enthalpies have been assessed also by molecular dynamics (MD) simulations. Verevkin et al. [7] reported experimental and MD-simulated vaporization enthalpies of a series of imidazolium-based ionic liquids. The latter were 20–30 kJ·mol^−1^ higher than the experimental values, which ranged from 120 to 190 kJ·mol^−1^. However, the MD-simulation results helped to explain the observed trends [7]. MD simulations resulted in fairly good values of vaporization enthalpies of water, ethanol, isooctane, methanol, hydrazine, chloroform, and acetone, although dependent on the force field models [8]. For example, the simulated vaporization enthalpy of water at boiling temperature varied from 39.6 ± 2.1 kJ·mol^−1^ (simulated *T*_b, sim_ = 344.7 ± 1.2 K) to 51.4 ± 1.0 kJ·mol^−1^ (*T*_b, sim_ = 397.7 ± 1.3 K) [8], while the value reported in critical tables is 40.65 kJ·mol^−1^ at 373.15 K [9]. Thus, the simulations still cannot replace the measurements.

Azeotropes are liquid mixtures whose components cannot be separated by fractional distillation because the vapor has the same composition as the liquid phase in equilibrium [10]. For this reason, the vaporization of pure liquids and azeotropes can be determined by applying similar experimental methods [11]. Despite that, reports of vaporization enthalpies of azeotropes are scarce. A search in databases, query “vaporization and (enthalpy or heat) and azeotrope”, resulted in a set of just 22 records in the Web of Science [12] and 36 in Scopus [13]. From those, only one referred to a recent paper about vaporization enthalpies of azeotropes determined experimentally at temperature 298.15 K [14]. Vaporization enthalpies of azeotropes were measured in the past, e.g., [11,15,16,17,18,19,20,21].

The new experiments in this field encouraged us to calculate vaporization enthalpies of azeotropes from the respective enthalpies of their pure components using the well-known thermochemical cycle method. Then we compared the calculation results with the measured vaporization enthalpies [11,15,16] and those derived from the enthalpies of solution [14]. We also suggested an approach to the uncertainty assessment based on the Monte Carlo simulation, which supplemented information gained from the classical calculus of errors with a piece of knowledge about the distribution of the calculation result.

We believe that our study may help to decide whether the expected uncertainty of the measured vaporization enthalpy of azeotrope justifies the experiments, being by their nature more demanding than simple calculations. In this paper, we focused attention on the uncertainty in the vaporization enthalpy of an azeotrope due to the calculation procedure. The measurement uncertainty was discussed only briefly, without an attempt at a comparison of the experimental methods in this respect.

## 2. Results

Vaporization enthalpies of binary azeotropes were calculated from the respective enthalpies of their pure components using the thermochemical cycles reported in Section 4 “Calculations”. The calculations were performed at the following three levels of approximation:

- Level 1. The heat effects of all processes were considered, except that of the mixing of gases, i.e., the excess enthalpy of the gaseous azeotrope was *H*^E^(g) = 0. The total enthalpy of vaporization of the azeotrope Δ_vap_*H*_Az_ at its boiling temperature *T*_Az_ was given by Equation (21). The boiling temperatures of the azeotropes differed from those of their pure components. A similar procedure with Equation (11) gives the vaporization enthalpies at *T*_0_ = 298.15 K.
- Level 2. The vaporization enthalpy of the azeotrope was the sum of the mole-fraction-weighted average of the vaporization enthalpies of its pure components and the excess enthalpy of the liquid azeotrope. The Δ_vap_*H*_Az_ was calculated from Equation (25) with *H*^E^(g) = 0.
- Level 3. Similar to those on Level 2 with another simplification: the excess enthalpy of the liquid azeotrope *H*^E^(l) = 0, Equation (26).

Note that the vaporization enthalpy of an azeotrope calculated using the thermochemical cycle without approximations must be equal to that measured directly within the measurement uncertainty limits. Such calculations could serve as a thermodynamic consistency test for the measurement results.

### 2.1. Validation of the Calculation Methods and Assessment of Uncertainty

Enthalpies of vaporization of twelve azeotropes at their boiling points were calculated at Levels 1 and 3 of the approximation Equations (21) and (26). In the calculations, the values of the vaporization enthalpies of pure compounds at their boiling points were used.

The calculation results for the boiling temperatures of the azeotropes along with the vaporization enthalpies measured calorimetrically [11,15,16] are reported in Table 1. Those reported in [11] were assumed to be the correct (“true”) values. Thus, the absolute and relative errors of the calculated vaporization enthalpies were defined as:

(4) $$∆=\Delta_{vap}H_{Az}(calc)-\Delta_{vap}H_{Az}\left(lit\right)$$

and

(5) $$\delta =\frac{\Delta }{\Delta_{vap}H_{Az}\left(lit\right)},$$

respectively, where Δ_vap_*H*_Az_(lit) refers to the value of the vaporization enthalpy measured directly [11].

The error in the vaporization enthalpies calculated from Equation (21) does not exceed 0.3 kJ·mol^−1^, i.e., the 1.1% of the measured value. This evidenced the thermodynamic consistency of the data and justified the approximations applied in the calculations. The weighted average approximation in Equation (26) proved to be surprisingly good. The respective maximum errors are 0.7 kJ·mol^−1^ and 2.1% of the measured value. In all cases, the vaporization enthalpies calculated from Equation (21) were larger than the measured ones, which probably resulted from the applied approximations. Only one value calculated from Equation (26), that for methanol–benzene azeotrope, was slightly smaller than the measured enthalpy. For this reason, the uncertainty interval would be asymmetrical for the calculated vaporization enthalpy, reflecting the tendency to slight overestimation of the latter. Based on these considerations, the uncertainty interval can be assessed at

(6) $${∆}_{vap}H_{Az}={∆}_{vap}H_{Az}(calc.)\frac{+0.0}{-0.5}(kJ\cdot {mol}^{-1})$$

for the values obtained from Equation (21), and

(7) $${∆}_{vap}H_{Az}={∆}_{vap}H_{Az}(calc.)\frac{+0.1}{-1.0}(kJ\cdot {mol}^{-1})$$

for those from Equation (26). Since the estimations are rather conservative, we believe that they are valid for the calculated Δ_vap_*H*_Az_ at *T*_0_ = 298.15 K too. Note that the above uncertainty intervals represent just the share due to the thermochemical cycle calculations in the total combined uncertainty of the azeotrope vaporization enthalpy. The latter depends also on the uncertainties in the vaporization enthalpies of pure components of the azeotrope. These are not discussed here as they are characteristic of the experimental method.

**Figure 1:**
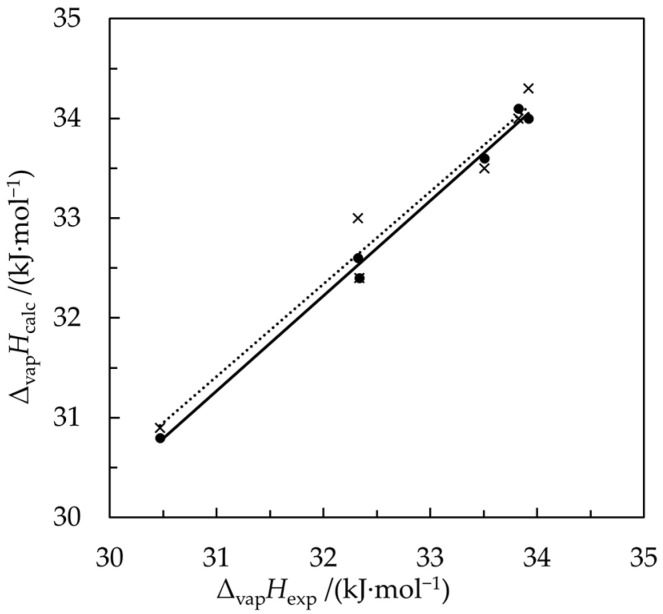
Vaporization enthalpies of azeotropes at their boiling temperatures *T*_Az_ measured directly, Δ_vap_*H*_exp_, [11] vs. the calculated values Δ_vap_*H*_calc_. Filled circles—Δ_vap_*H*_calc_ calculated using the thermochemical cycle Equation (21); crosses—Δ_vap_*H*_calc_ approximated by weighted averages of the vaporization enthalpies of pure components using Equation (26); solid and broken lines—respective regression lines fitted by the least squares method.

Figure 1 illustrates the good agreement of the calculated results with the experimental vaporization enthalpies. The simple regression lines were fitted using the Statistica program [22]. The values of the slope, 0.952 ± 0.039, and intercept, 1.77 ± 1.27, of the regression line for the Δ_vap_*H*_Az_ given by Equation (21) do not differ from 1 and 0, respectively, at the confidence level of 95%. For the Δ_vap_*H*_Az_ calculated from Equation (26), the results of the fitting are similar: The slope, 0.926 ± 0.090, and intercept, 2.7 ± 3.0 do not differ from 1 and 0 at the same confidence level.

The differences between the vaporization enthalpies calculated from Equations (21) and (26) are indeed small. This can be explained by the partial compensation of the heat effects of the mixture separation and heating and cooling of the system. The effects related to the vaporization enthalpy of the azeotrope were defined as:

(8) $$\delta_{Sep}\left(T\right)=\frac{{-H}^{E}}{{∆}_{vap}H_{Az}\left(T\right)}$$

for the separation, and

(9) $$\delta_{HC}\left(T\right)=\frac{{∆}_{i2}H+{∆}_{14}H+{∆}_{i7}H}{{∆}_{vap}H_{Az}\left(T\right)}$$

for the heating and cooling. In Equations (8) and (9), *T* = *T*_0_ and *i* = 1 for the thermochemical cycle in Figure 5, while *T* = *T*_Az_ and *i* = 2 for that in Figure 6. The values of *δ*_Sep_ and *δ*_HC_ are reported in Table 2. Negative numbers indicate that neglecting the heat of separation results in an estimation lower than the true value. Positive numbers for heating and cooling have the opposite meaning. The maximum combined effect of the three processes at *T*_Az_ is just −1.4% of Δ_vap_*H*_Az_ for the benzene–1-propanol azeotrope. Note that all the mixtures compared here show positive excess enthalpies. Such a compensation for the heat effects would not occur for the systems with negative *H*^E^. It is obvious that the heating and cooling of the system has a greater share of the estimated vaporization enthalpy at *T*_0_ = 298.15 K because the temperature difference ($T_{B}-T_{0}$) is greater than ($T_{B}-T_{Az}$). Nevertheless, it seldom exceeds 9% of Δ_vap_*H*_Az_.

### 2.2. Comparison of the Calculated Vaporization Enthalpies with Those Obtained from the Enthalpies of Solution

The calculation methods, validated in the manner described in Section 2.1, were applied for twelve azeotropes whose vaporization enthalpies were reported in [14]. In the latter study, the indirect method based on the measured solution enthalpy and molar refraction was applied Equations (2) and (3). The results for *T* = 298.15 K are compared in Table 3.

The values of Δ and *δ* in Table 3, calculated from Equations (4) and (5), are not errors but rather absolute and relative differences between the enthalpies obtained by solution calorimetry method with cyclohexane solvent [14] and those calculated from Equations (11) and (26), which were the reference values. We have chosen the results for cyclohexane rather than *n*-heptane solvent because they are slightly closer to the calculated vaporization enthalpies. The conclusions for the two datasets reported in [14] would be the same with minor quantitative differences.

The values of the azeotrope vaporization enthalpies calculated using Equation (25) with *H*^E^(g) = 0 are 0.4 to 0.7 kJ·mol^−1^ higher than those obtained from the thermochemical cycle Equation (11) for the (benzene or cyclohexane)–alcohol and acetonitrile–benzene azeotropes. For the *n*-hexane–1-butanol azeotrope they are lower by 0.1 kJ·mol^−1^.

Unfortunately, the vaporization enthalpies of the azeotropes measured directly at the temperature of 298.15 K were unavailable for comparison. For this reason, we extrapolated the experimental results of Svoboda et al. [11] to this temperature assuming a linear dependence of the vaporization enthalpy on temperature. Residual analysis of the fits and high values of the determination coefficients proved that the assumption was reasonable. The extrapolated enthalpies were only slightly higher than those measured at the temperatures closest to 298.15 K, by 0.18 to 1.49 kJ·mol^−1^. These vaporization enthalpies at *T* = 298.15 K with characteristics of the fits are reported in Table 4.

**Figure 2:**
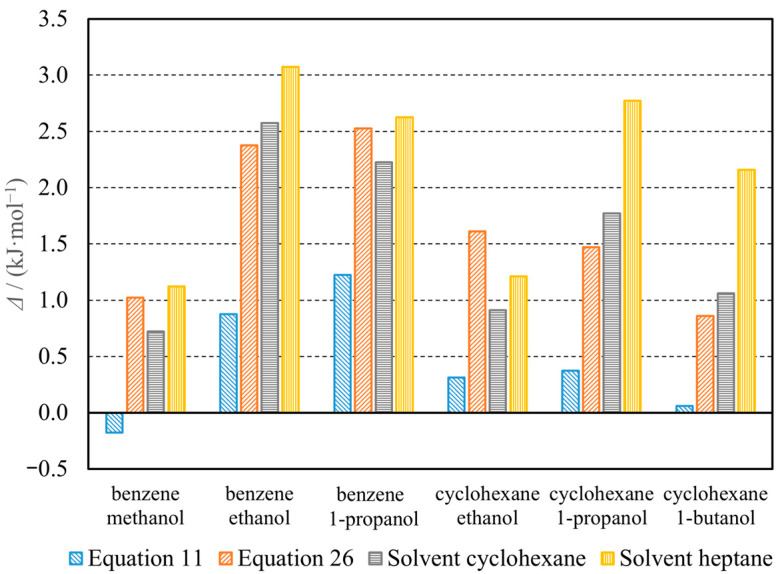
Differences between the vaporization enthalpies calculated in this work using Equations (11) and (26), those assessed from the enthalpies of solution in cyclohexane and heptane [14], and the reference values of the vaporization enthalpies reported in Table 4.

Figure 2 shows the differences between the vaporization enthalpies obtained by various methods and calculated from a formula analogous to Equation (4) where the reference (“true”) values were those reported in Table 4. The thermochemical cycle-based Equation (11) seems to provide better results than the solution calorimetry method suggested in [14]. Each vaporization enthalpy obtained from Equation (11) was closer to the reference value than the corresponding value obtained from the solution enthalpy. Its relative deviation was within the limits of −0.5 to 3.6%. The mole-fraction-weighted averages from Equation (26) allow for at least an equally good assessment of the true vaporization enthalpies as the solution calorimetry. In general, calculations with Equation (26) and the solution calorimetry method with cyclohexane and heptane as the solvents [14] overestimated the azeotrope vaporization enthalpies by 2.6–7.4%, 2.1–7.3%, and 3.2–8.7%, respectively.

The azeotropes vaporization enthalpies reported in [14] are larger than those calculated from Equation (11) by 0.6 to 5.5 kJ·mol^−1^ (1.6 to 14.8%). A substantial discrepancy of 14.8% is observed between the vaporization enthalpies of the *n*-hexane–1-butanol azeotrope.

Smaller differences exist between the vaporization enthalpies reported in [14] and those calculated as the mole-fraction-weighted averages of the enthalpies for pure components using Equation (26). Only three out of twelve compared Δ_vap_*H*_Az_ values show discrepancies exceeding the combined uncertainties of the measurement and calculation (Table 3). However, the three are huge in comparison with the other ones, varying from 3.5 to 5.3 kJ·mol^−1^, i.e., from 9.2 to 14.2%. It seems that the solution enthalpy method failed in the case of the *n*-hexane-containing azeotropes. The following reasoning supports this supposition.

Let us assume that the vaporization enthalpy for the *n*-hexane–1-butanol azeotrope reported in [14], 37.2 kJ·mol^−1^ at 298.15 K, is correct. The mole-fraction-weighted average of the vaporization enthalpies of this mixture components is 31.9 kJ·mol^−1^ (Table 3). It follows from the thermochemical cycle results that the difference between the excess molar enthalpies of the liquid and gaseous mixtures is equal to (31.9−37.2 = −5.3) kJ·mol^−1^. Thus, the excess molar enthalpy of the liquid mixture would be huge and negative, at least –5.3 kJ·mol^−1^, while it is positive, ca. +0.23 kJ·mol^−1^ [23]. That is because the endothermic breaking of H-bonds in the alcohol accompanies its mixing with the hydrocarbon. Note also that there is just ca. 5% by mole of alcohol in the azeotrope [14]. Indeed, such a small amount of alcohol cannot raise the vaporization enthalpy to 37.2 kJ·mol^−1^ from that of 31.0 ± 1.0 kJ·mol^−1^ [24] for pure hexane. For the two other *n*-hexane azeotropes, the results are similar albeit less spectacular.

**Figure 3:**
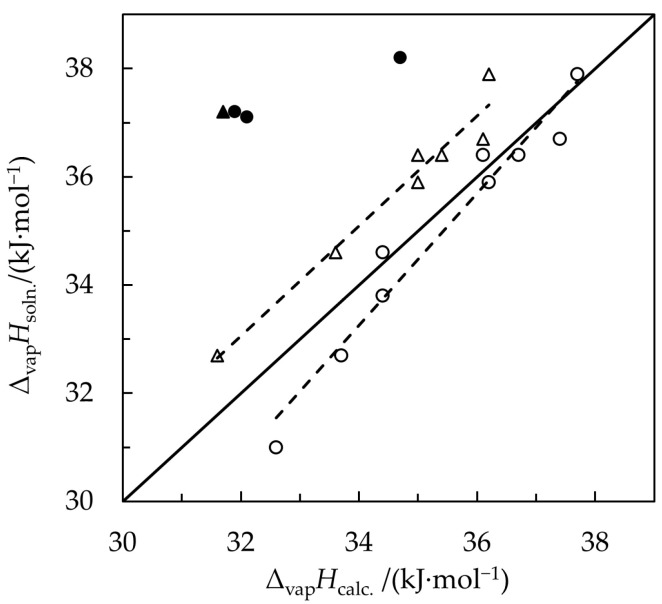
Vaporization enthalpies of azeotropes at *T*_0_ = 298.15 K calculated in this work, Δ_vap_*H*_calc._, vs. the values obtained from the enthalpies of solution [14], Δ_vap_*H*_soln_. Triangles—enthalpies calculated using the thermochemical cycle Equation (11); circles—enthalpies approximated by weighted averages of the vaporization enthalpies of pure components using Equation (26); broken lines—regression lines fitted by the least squares method. The solid line represents a hypothetical ideal case where ${∆}_{vap}H_{soln.}={∆}_{vap}H_{calc.}$; the filled triangle and circles—outliers for azeotropes with *n*-hexane that were not included in the regression line fittings.

The regression lines plotted in Figure 3 illustrate systematic differences between the solution calorimetry results [14] and the vaporization enthalpies calculated from Equations (11) and (26). Although the agreement seems better for Δ_vap_*H*_Az_ calculated from the approximate Equation (26), the differences are not constant but rather increase with decreasing vaporization enthalpies. The close agreement between the vaporization enthalpies measured directly and those calculated using thermochemical cycles reported in Section 2.1 of this paper, along with the discussion of molecular interactions in the previous paragraph, suggest that the results for *n*-hexane-containing azeotropes obtained through the solution calorimetry method are systematically higher than expected. However, the reasons behind the failure of the solution enthalpy method applied to these systems are unclear. We are unfamiliar with the solution calorimetry approach, so we simply acknowledge this issue without speculating on potential sources of error.

### 2.3. Monte Carlo Simulation of the Combined Uncertainty

Probably the most important question is how good the expected result must be to justify the effort of measurements of the vaporization enthalpy of an azeotrope. Indeed, the thermochemical cycle calculations are much less time-consuming and simpler than any of such experiments. Thus, the experiments are unnecessary unless the measured values are more nearly accurate than the calculated ones.

Usually, two groups of methods are applied in assessments of uncertainty: the classical calculus of errors and statistical methods [25]. The calculus of errors is applied in the planning stage of the experiments to facilitate the decision of whether the expected maximum uncertainty satisfies expectations. In this approach, the maximum error is calculated based on the assumption about the error additivity. For example, the maximum error of the Δ_vap_*H*_Az_ calculated from Equation (25) is given by:

(10) $$\Delta_{\chi_{Az}}=\left|\chi_{A}^{o}-\chi_{B}^{o}\right|\Delta_{x_{A}}+x_{A}{∆}_{\chi_{A}^{o}}+\left(1-x_{A}\right){∆}_{\chi_{B}^{o}}+{∆}_{H^{E}(l)}+{∆}_{H^{E}(g)},$$

where the symbol *χ* stands for the vaporization enthalpy, ${\chi =∆}_{vap}H$, and it is used here just to make the formula readable. Functions for pure substances are marked with superscript “o”. The Δ_vap_*H*_Az_ of the ethanol–benzene azeotrope at *T*_0_ = 298.15 K calculated from Equations (10) and (25) is 36.89 ± 0.34 kJ·mol^−1^. It differs from 36.2 kJ·mol^−1^ reported in Table 3 because the latter was calculated from Equation (11). In the calculations, we applied the uncertainties of the vaporization enthalpies of pure ethanol and benzene reported in Table 6. The others we assumed to be 0.005 for *x*_A_, and 0.05 and 0.01 kJ·mol^−1^ for the excess enthalpies of the liquid and gaseous azeotrope, respectively.

However, errors rarely propagate in such an adverse way [25]. For a more realistic assessment, we performed a simple Monte Carlo simulation of the vaporization enthalpy of the ethanol–benzene azeotrope at *T*_0_ = 298.15 K. The simulation consisted of 1 · 10^6^ trials. In each trial, Δ_vap_*H*_Az_ was calculated from Equation (25) using the values of Δ_vap_*H*_A_°, Δ_vap_*H*_B_°, *H*^E^(l), and *H*^E^(g) chosen randomly from their ranges defined as continuous uniform distributions. The latter results from the principles of the calculus of errors, which can be used for the estimation of errors of a single or even a hypothetical measurement [25].

**Figure 4:**
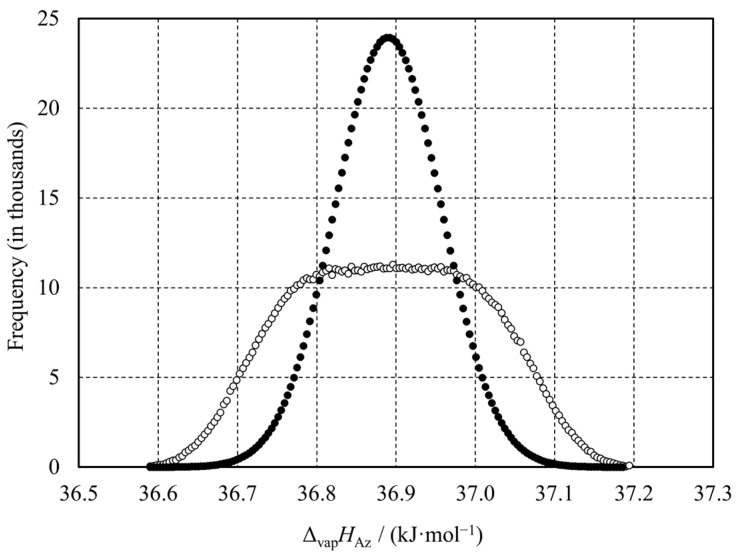
Distributions of the vaporization enthalpy of the ethanol–benzene azeotrope at *T*_0_ = 298.15 K. Open symbols: the result of Monte Carlo simulation consisting of 1 · 10^6^ trials. Each point represents the number of simulation results in one of the 150 equal-width intervals within the total uncertainty range of 36.89 ± 0.34 kJ·mol^−1^. Closed symbols: population in the same intervals calculated from the normal distribution equation with the standard deviation of 0.067 kJ·mol^−1^.

The resulting distribution of the Δ_vap_*H*_Az_, together with the Gaussian function obtained in the manner described in the next paragraph, is plotted in Figure 4. Indeed, the distribution of Δ_vap_*H*_Az_ is not uniform. The ranges of the assessed intervals of the Δ_vap_*H*_Az_ values for several confidence levels are reported in Table 5. For the commonly accepted 95% confidence level, the uncertainty is ±0.21 kJ·mol^−1^, which is ca. 62% of the maximum error estimated using Equation (10).

We may argue that one assumes normal distribution rather than uniform in experimental practice. However, researchers seldom state a type of statistical distribution explicitly, but report only uncertainty. Assuming that the uncertainties of Δ_vap_*H*_A_°, Δ_vap_*H*_B_°, *H*^E^(l), and *H*^E^(g) from the previous paragraph were triple the values of respective standard deviations, the standard deviation of the Δ_vap_*H*_Az_ could be calculated using statistical methods rather than the calculus of errors (e.g., [25]). In this case, the Monte Carlo simulation was not necessary. In this approach, the squared partial derivatives and variances (i.e., squared standard deviations) substitute the moduli of the partial derivatives and the maximum errors, respectively, in Equation (10). The resulting standard deviation of Δ_vap_*H*_Az_ is 0.067 kJ·mol^−1^. Figure 4 shows the frequency graph rescaled to match the Monte Carlo results for 10^6^ trials. The tripled standard deviation, 0.201 kJ·mol^−1^, can be identified with the “total uncertainty”. Indeed, the latter is smaller than the maximum error assessed for 0.34 kJ·mol^−1^ using the calculus of errors.

The above reasoning evidenced that the classical calculus of errors resulted in a rather conservative estimation of the calculation uncertainty. Thus, the uncertainty range of the expected experimental Δ_vap_*H*_Az_ value must be substantially narrower than the latter. Otherwise, the measurements are not worth undertaking. Note that the uncertainty of the calculated Δ_vap_*H*_Az_ reported in this example cannot be treated as a reference value. The calculation uncertainty should be assessed individually for each azeotrope before measurements.

## 3. Discussion

The calculated vaporization enthalpies at the boiling temperature of the six azeotropes studied were on average just 0.2 kJ·mol^−1^ higher than those measured directly by the calorimetric method, reported in [11]. This high accuracy was achieved despite the simplifying assumptions of the thermodynamic ideal gas phase and the constant heat capacities of the azeotrope components. In the most unfavorable case of the cyclohexane–1-butanol system, the relative error was 1.1%. Moreover, the mole-fraction-weighted averages of the vaporization enthalpies of the pure components using Equation (26) also gave plausible approximations of Δ_vap_*H*_Az_. Indeed, the maximum calculation error was 0.7 kJ·mol^−1^, which was 2.1% of the measured value (Table 1).

The comparison with the data reported in [11,15,16] evidenced that simple thermochemical calculations allowed for the assessment of the azeotrope vaporization enthalpy with similar uncertainty to that attainable in experiments. Indeed, the values of the combined heat effects of heating/cooling and mixing/de-mixing of the azeotrope components are often close to the declared measurement uncertainties because the enthalpies of mixing are much smaller than the vaporization enthalpies. An explanation in terms of molecular theory is obvious: The molecular attraction in the gas phase is weaker than in liquids due to much larger intermolecular distances in the former. Thus, any method of determination of Δ_vap_*H*_Az_ should provide more nearly accurate results than the thermochemical cycle calculations do. This is especially important for methods based on empirical relationships, e.g., that of solution calorimetry [14], and those applying model calculations, such as NRTL or UNIFAC [26].

The uncertainty of the thermochemical calculations assessed by the classical calculus of errors may be treated as a guide to whether measurements are worthy of making. However, such an estimation is rather conservative and does not inform about the statistical distribution of the calculated vaporization enthalpy of the azeotrope. For this reason, the Monte Carlo simulation suggested in Section 2.3. of this work provided a more realistic uncertainty interval of the calculated Δ_vap_*H*_Az_. Results of such simulations can be helpful in analyses of thermodynamic consistency of the vaporization enthalpies of pure liquids and their azeotropic mixtures. Concurrently, the uncertainty of the calculated Δ_vap_*H*_Az_ function can be assessed using the statistical methods provided that its arguments, such as Δ_vap_*H*_A_°, Δ_vap_*H*_B_°, *H*^E^(l), *H*^E^(g), etc., are distributed normally. Correctly estimated uncertainty of the calculations is especially important for the methods based on empirical correlations rather than strict thermodynamic equations.

## 4. Calculations

### 4.1. General Thermochemical Cycle

**Figure 5:**
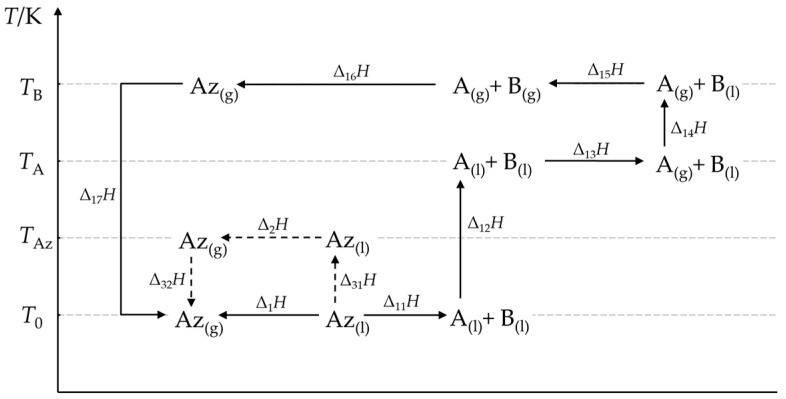
Thermochemical cycle for calculating the vaporization enthalpy of a positive azeotrope. Δ_1_*H* is the vaporization enthalpy at the reference temperature *T*_0_; Δ_2_*H* is the vaporization enthalpy at the boiling temperature of the azeotrope *T*_Az_. The remaining symbols were explained after Equation (11) in the text.

Enthalpies of vaporization of binary azeotropes Δ_vap_*H*_Az_ can be calculated from the respective enthalpies of pure components in the mixtures, their heat capacities in liquid and gaseous phases, and the enthalpies of mixing (i.e., the excess enthalpies) for the two phases. In this manner, it is possible to determine the vaporization enthalpy for any temperature, the boiling temperature *T*_Az_ in particular, by applying a proper thermochemical cycle. Such a cycle for a positive azeotrope is depicted in Figure 5. In this work, the mixture components are denoted A and B. Component A has a lower boiling temperature than Component B. In this paper, the reference temperature *T*_0_ = 298.15 K for consistency with common practice.

The enthalpy Δ_1_*H* refers to the reference temperature *T*_0_. It is equal to the sum of the component enthalpies according to the following equation:

(11) $${{∆}_{vap}H_{Az}(T_{0})=∆}_{1}H=\sum_{i=1}^{7}{∆}_{1i}H,$$

where

(12) $${∆}_{11}H=-H^{E}$$

(separation of the liquid mixture),

(13) $${∆}_{12}H=\int_{T_{0}}^{T_{A}}(C_{p,A}^{o}(l)\cdot x_{A}+C_{p,B}^{o}(l)\cdot x_{B})dT$$

(heating the liquids A and B to the boiling temperature of A),

(14) $${∆}_{13}H=x_{A}\cdot {∆}_{vap}H_{A}^{o}$$

(vaporization of A),

(15) $${∆}_{14}H=\int_{T_{A}}^{T_{B}}\left(C_{p,A}^{o}(g\right)\cdot x_{A}+C_{p,B\;}^{o}(l)\cdot x_{B})dT$$

(heating gaseous A and liquid B to the boiling temperature of B),

(16) $${∆}_{15}H=x_{B}\cdot {∆}_{vap}H_{B}^{o}$$

(vaporization of B),

(17) $${∆}_{16}H=H^{E}(g)$$

(mixing gaseous A with B), and

(18) $${∆}_{17}H=\int_{T_{B}}^{T_{0}}\left(C_{p,A}^{o}(g)\cdot x_{A}+C_{p,B}^{o}(g)\cdot x_{B}\right)dT\;$$

(cooling the gaseous mixture).

In the above equations, *x* is the mole fraction, *T*_0_ is the reference temperature, e.g., 298.15 K, *T* is the boiling temperature, *C_p_* is the molar isobaric heat capacity, Δ_vap_*H* is the vaporization enthalpy, *H*^E^ is the excess enthalpy, letters “l” and “g” in parentheses stand for “liquid” and “gas”, and superscript “o” and subscripts A, B, and Az denote the “pure substance”, the two mixture components, and the azeotrope, respectively.

### 4.2. Simplified Equations for Non-Isothermal Conditions

A difference between the vaporization enthalpy of the azeotrope measured directly and that calculated from the thermochemical cycle results solely from the experimental errors of particular quantities. However, several simplifications could be applied because of practical reasons. First, the enthalpy of mixing for the gaseous components is hardly ever measured. Fortunately, it is approximately equal to zero for real gases, and even equal to zero for the ideal one. The ideal gas approximation results in another simplification: The molar isobaric heat capacity of the gaseous azeotrope is equal to the weighted average of molar isobaric heat capacities of pure gases A and B:

(19) $$C_{p,Az}(g)=C_{p,A}^{o}(g)\cdot x_{A}+C_{p,B}^{o}(g)\cdot x_{B}.$$

An analysis of the enthalpy shares in Δ_vap_*H*_Az_ defined by Equations (12)–(18) and reported in Section 2.1. of this work evidenced that heating and cooling of the system Equations (13), (15), and (18) contribute by less than 10% of the total enthalpy change. Thus, the averaged values of the heat capacities for the whole temperature range, ${\bar{C}}_{p}$, could be applied rather than functional relationships *C_p_*(*T*) without the risk of substantially worsening calculational accuracy. Consequently, the integrals in Equations (13), (15), and (18) could be substituted by the approximate equations of the form:

(20) $$\Delta H=\int_{T_{1}}^{T_{2}}C_{p}dT\approx {\bar{C}}_{p}\left(T_{2}-T_{1}\right).$$

**Figure 6:**
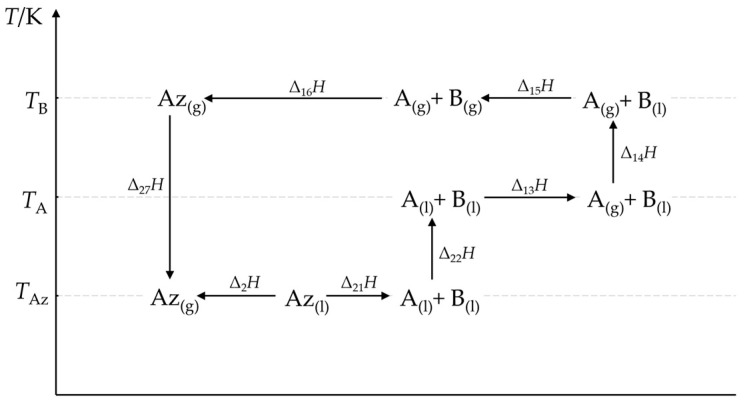
Thermochemical cycle for calculating the vaporization enthalpy Δ_2_*H* of a positive azeotrope at its boiling temperature.

The enthalpy of vaporization of the azeotrope at its boiling point (*T*_Az_) can be calculated using the thermochemical cycle reported in Figure 6. The calculation principle is the same as in the previous example, except the vaporization temperature of the azeotrope is *T*_Az_ rather than *T*_0_, and:

(21) $${∆}_{vap}H_{Az}\left(T_{Az}\right)=\sum_{j=3}^{6}{∆}_{1j}H+\sum_{j=1}^{2}{∆}_{2j}H+{∆}_{27}H.$$

The values of Δ_1*j*_*H* in Equation (21) are calculated from Equations (14)–(17), while those of the remaining Δ_2*j*_*H* in the following way:

(22) $${∆}_{21}H\approx {∆}_{11}H=-H^{E},$$

(23) $${∆}_{22}H=\int_{T_{Az}}^{T_{A}}(C_{p,A}^{o}(l)\cdot x_{A}+C_{p,B}^{o}(l)\cdot x_{B})dT,$$

(24) $${∆}_{27}H=\int_{T_{B}}^{T_{Az}}\left(C_{p,A}^{o}(g)\cdot x_{A}+C_{p,B}^{o}(g)\cdot x_{B}\right)dT\;.$$

The difference between Δ_21_*H* (Equation (22)) and Δ_11_*H* (Equation (12)) results from the different heat capacities of the liquid and gaseous azeotrope, which are illustrated by the dashed line arrows in Figure 5. This approximation is necessary if the excess enthalpies at the boiling temperature are unavailable. The integrals in Equations (23) and (24) can be approximated by formulas in the form of Equation (20).

### 4.3. Simplified Equations for Isothermal and Quasi-Isothermal Conditions

If the vaporization enthalpy of the azeotrope is to be calculated for a temperature for which the vaporization enthalpies of its pure components are known, then the following Equation (25) is suitable:

(25) $${{∆}_{vap}H_{Az}=x}_{A}\cdot {∆}_{vap}H_{A}^{o}+x_{B}\cdot {∆}_{vap}H_{B}^{o}-H^{E}(l)+H^{E}(g).$$

Since the heat effects of mixing and de-mixing of the systems are much smaller than the vaporization enthalpies and they partially cancel each other out, an approximate formula for the vaporization enthalpy of the azeotrope in isothermal conditions can be used:

(26) $${{∆}_{vap}H_{Az}\approx x}_{A}\cdot {∆}_{vap}H_{A}^{o}+x_{B}\cdot {∆}_{vap}H_{B}^{o}.$$

In the calculations, we used the values of Δ_vap_*H*_A_° and Δ_vap_*H*_B_° at *T* = 298.15 K. Equation (26) can be also applied to the vaporization enthalpies at the boiling temperatures. In this case, the calculated Δ_vap_*H* value does not include the heat effects due to the different boiling temperatures of the pure components and the mixture. This approach is justified by the fact that the boiling temperatures do not differ significantly from one another and the heat effects of heating and cooling are quite small and partially cancel each other out. For this reason, we called these conditions “quasi-isothermal”. The latter method leads to worse results than the previously discussed ones.

### 4.4. Data Used in the Calculations

The majority of literature data, including boiling temperatures (*T*_boil_) and vaporization enthalpies of pure components Δ_vap_*H*° at *T*_0_ = 298.15 K, were taken from the NIST Chemistry WebBook [24]. The data for pure substances are collected in Table 6.

The azeotrope compositions from [14] were used in the calculations. They slightly differ from the values reported in other sources, but this does not influence the calculation results significantly. The data for azeotropes are collected in Table 7. Because of the lack of data for the isobaric heat capacity of the gaseous acetonitrile, the value of *C_p_*°(g) of benzene was applied to the gas phases of the benzene–acetonitrile azeotrope.

Because of the incomplete data for four azeotropes, i.e., acetonitrile–ethyl acetate, acetonitrile–tetrachloromethane, *n*-hexane–ethanol, and *n*-hexane–1-propanol, Equations (11) and (21) could not be applied to the calculations for these systems. In these cases, only simplified calculations using Equation (26) were possible.

## 5. Conclusions

Thermochemical cycles applied in this work are suitable for calculations of the vaporization enthalpies of the azeotropes. The Δ_vap_*H*_Az_ values calculated from a thermochemical cycle without approximations, such as those illustrated in Figure 5 and Figure 6, are indeed equal to the values measured directly within the measurement uncertainty range. This trivial conclusion results from the properties of enthalpy as a function of state. More interesting from the practical point of view was whether the vaporization enthalpies of the azeotropes calculated with simplified formulae were still acceptable. The following two approximations did not significantly influence the calculation results: (*i*) The gas phase was the thermodynamic ideal mixture of perfect gases, and (*ii*) the heat capacities of the liquid and gaseous phases were constant in the discussed interval of temperature. The calculation error due to these simplifications was close to the measurement uncertainty attainable in the direct calorimetric measurements. The relative error was 1.1% in the most unfavorable case.

Vaporization enthalpies of azeotropes can also be approximated by the mole-fraction-weighted averages of the respective enthalpies of their pure components Equation (26). The calculated Δ_vap_*H*_Az_ did not differ much from those obtained in the calorimetric experiment, with a relative error of up to 2.1%. Experimental methods based on empirical relationships rather than strict thermodynamic relationships may not give better results than this simple method, as was evidenced by the comparison with Δ_vap_*H*_Az_ obtained by the solution calorimetry [14].

In general, Δ_vap_*H*_Az_ is worthy of calculation using Equations (11), (21), and (25), or even Equation (26), before undertaking the measurements. The maximum error of the calculation result can be assessed by the classical calculus of errors. The expected measurement error must be substantially lower. A Monte Carlo simulation, similar to that suggested in this work, may help in setting a reasonable limit for the measurement error.

## Figures and Tables

**Table 1:** Vaporization enthalpies Δ_vap_*H*_Az_ of binary azeotropes at their boiling points *T*_Az_, calculated from Equations (21) and (26), the measurement results reported in the literature [11,15,16], and calculation errors: absolute, Δ, and relative, *δ*, defined by Equations (4) and (5). Some enthalpies could not be calculated from Equation (21) because of the lack of necessary data.

Azeotrope	Measured Δ_vap_*H*_Az_/ (kJ∙mol^−1^)	Thermochemical Cycle, Equation (21)			Weighted Average, Equation (26)		
		Δ_vap_*H*_Az_/ (kJ∙mol^−1^)	Δ/ (kJ∙mol^−1^)	*δ*/%	Δ_vap_*H*_Az_/ (kJ∙mol^−1^)	Δ/ (kJ∙mol^−1^)	*δ*/%
Benzene–methanol	33.505 ^a^ 33.64 ^b^	33.6	0.1	0.3	33.499 ^d^	−0.006	−0.02
Benzene–ethanol	33.920 ^a^ 34.34 ^b^	34.0	0.1	0.2	34.3	0.4	1.1
Benzene–1-propanol	32.321 ^a^ 31.89 ^b^	32.6	0.3	0.9	33.0	0.7	2.1
Cyclohexane–ethanol	33.823 ^a^ 34.75 ^c^	34.1	0.3	0.8	34.0	0.2	0.5
Cyclohexane– 1-propanol	32.334 ^a^ 34.04 ^c^	32.4	0.1	0.2	32.4	0.1	0.2
Cyclohexane–1-butanol	30.468 ^a^ 32.36 ^c^	30.8	0.3	1.1	30.9	0.4	1.4
Acetonitrile–benzene	-	30.1	-	-	30.3	-	-
Acetonitrile– ethyl acetate	-	-	-	-	31.2	-	-
Acetonitrile– tetrachloromethane	-	-	-	-	29.8	-	-
*n*-Hexane–ethanol	-	-	-	-	32.0	-	-
*n*-Hexane–1-propanol	-	-	-	-	29.8	-	-
*n*-Hexane–1-butanol	-	29.3	-	-	29.4	-	-

^a^ Reference [11]. ^b^ Reference [15]. ^c^ Reference [16]. ^d^ Three decimal digits to allow for the comparison with the measured value.

**Table 2:** Relative contributions of the mixture separation, *δ*_sep_, and the heating and cooling of the system, *δ*_HC_, to the vaporization enthalpies of the azeotropes at the reference temperature *T*_0_ = 298.15 K and the boiling temperature *T*_Az_, calculated for the thermochemical cycles reported in Figures 5 and 6.

Azeotrope	*T*_0_		*T*_Az_	
	*δ*_sep_/%	*δ*_HC_/%	*δ*_sep_/%	*δ*_HC_/%
Benzene–methanol	−1.5	5.8	−1.6	1.8
Benzene–ethanol	−2.3	7.6	−2.4	1.7
Benzene–1-propanol	−2.4	9.1	−2.6	1.2
Cyclohexane–ethanol	−1.8	7.4	−1.9	2.1
Cyclohexane–1-propanol	−1.6	8.9	−1.7	1.6
Cyclohexane–1-butanol	−1.1	9.2	−1.2	0.8
*n*-Hexane–1-butanol	−0.9	8.1	−1.0	0.6
Acetonitrile–benzene	−1.4	5.7	−1.5	0.8

**Table 3:** Vaporization enthalpies Δ_vap_*H*_Az_ of binary azeotropes at the reference temperature *T*_0_ = 298.15 K calculated from Equation (11) (with *H*^E^(g) = 0) and (26) compared with those reported in [14], and the absolute, Δ, and relative, *δ*, differences Equations (4) and (5). Some enthalpies could not be calculated from Equation (11) because of the lack of necessary data.

Azeotrope	Δ_vap_*H*_Az_/(kJ∙mol^−1^) [14]		Thermochemical Cycle, Equation (11)			Weighted Average, Equation (26)		
	Solvent C_6_H_12_	Solvent C_7_H_16_	Δ_vap_*H*_Az_^e^/ (kJ∙mol^−1^)	Δ/ (kJ∙mol^−1^)	*δ*/%	Δ_vap_*H*_Az_^f^/ (kJ∙mol^−1^)	Δ/ (kJ∙mol^−1^)	*δ*/%
Benzene–methanol	35.9 ^a^	36.3 ^c^	35.0	0.9	2.5	36.2	−0.3	−0.8
Benzene–ethanol	37.9 ^b^	38.4 ^d^	36.2	1.7	4.5	37.7	0.2	0.5
Benzene–1-propanol	36.4 ^a^	36.8 ^c^	35.4	1.0	2.7	36.7	−0.3	−0.8
Cyclohexane–ethanol	36.7 ^b^	37.0 ^d^	36.1	0.6	1.6	37.4	−0.7	−1.9
Cyclohexane– 1-propanol	36.4 ^a^	37.4 ^c^	35.0	1.4	3.8	36.1	0.3	0.8
Cyclohexane–1-butanol	34.6 ^a^	35.7 ^c^	33.6	1.0	2.9	34.4	0.2	0.6
Acetonitrile–benzene	32.7 ^a^	32.8 ^c^	31.6	1.1	3.4	33.7	−1.0	−3.1
Acetonitrile– ethyl acetate	33.8 ^a^	33.6 ^c^	-	-	-	34.4	−0.6	−1.8
Acetonitrile– tetrachloromethane	31.0 ^a^	31.4 ^c^	-	-	-	32.6	−1.6	−5.2
*n*-Hexane–ethanol	38.2 ^a^	37.7 ^c^	-	-	-	34.7	3.5	9.2
*n*-Hexane–1-propanol	37.1 ^a^	38.1 ^c^	-	-	-	32.1	5.0	13.5
*n*-Hexane–1-butanol	37.2 ^a^	36.2 ^c^	31.7	5.5	14.8	31.9	5.3	14.2

The uncertainties (in kJ∙mol^−1^) are ^a^ ±0.9; ^b^ ±1.0; ^c^ ±1.4; ^d^ ±1.5; ^e^ +0.0/−0.5; ^f^ +0.1/−1.0.

**Table 4:** Extrapolated vaporization enthalpies Δ_vap_*H*_Az_ of binary azeotropes at the reference temperature *T*_0_ = 298.15 K calculated from the experimental data reported by Svoboda et al. [11] with the corrected coefficients of determination *R*^2^ and numbers of experimental points *n*.

Azeotrope	Δ_vap_*H*_Az_^a^/ (kJ∙mol^−1^)	Temperature Range ^b^ (K)	*R* ^2^	*n*
Benzene–methanol	35.18 ± 0.11	302.01–330.85	0.93	8
Benzene–ethanol	35.33 ± 0.27	324.17–340.85	0.80	6
Benzene–1-propanol	34.17 ± 0.60	318.15–349.45	0.99	6
Cyclohexane–ethanol	35.79 ± 0.10	321.67–338.15	0.98	6
Cyclohexane–1-propanol	34.63 ± 0.46	328.15–347.55	0.82	5
Cyclohexane–1-butanol	33.54 ± 0.18	323.15–352.55	0.98	5

^a^ Values with standard errors; the latter are not the measurement uncertainties but rather illustrate the quality of the fit. Svoboda et al. [11] reported the relative standard deviations of the mean vaporization enthalpies at constant temperature “lower than 0.2%”. ^b^ Temperature range of the measurements.

**Table 5:** The Monte-Carlo-simulated ranges of the calculated vaporization enthalpy of the ethanol–benzene azeotrope at *T*_0_ = 298.15 K.

Confidence Level	Δ_vap_*H*_Az_/(kJ·mol^−1^)	
	Minimum	Maximum
1	36.55	37.23
0.95	36.68	37.10
0.90	36.71	37.07

**Table 6:** Boiling temperatures *T*_boil_, vaporization enthalpies Δ_vap_*H*° at *T*_0_ = 298.15 K and *T*_boil_, and molar isobaric heat capacities of liquids *C_p_*°(l) and gases *C_p_*°(g) for the components of the binary azeotropes studied.

Substance	*T*_boil_/K	$\Delta_{\mathrm{vap}}H{}^{\circ}(T_{0})$ (kJ∙mol^−1^)	Δ_vap_*H*°(*T*_boil_)/ (kJ∙mol^−1^)	*C*_p_°(l)/ (J∙K^−1^∙mol^−1^)	*C*_p_°(g)/ (J∙K^−1^∙mol^−1^)
Methanol	337.8 ± 0.3 ^a^	37.6 ± 0.5 ^a^	35.21 ^b^	81.11 ^c^	44.06 ± 0.03 ^j^
Ethanol	351.5 ± 0.2 ^a^	42.3 ± 0.4 ^a^	38.55 ^b^	112.30 ^d^	65.21 ± 0.14 ^j^
1-Propanol	370.3 ± 0.5 ^a^	47.0 ± 1.0 ^a^	41.45 ^b^	143.78 ^d^	85.56 ± 0.14 ^j^
1-Butanol	390.6 ± 0.8 ^a^	52.0 ± 3.0 ^a^	43.32 ^b^	176.67 ^e^	108.03 ± 0.25 ^j^
*n*-Hexane	341.9 ± 0.3 ^a^	31.0 ± 1.0 ^a^	28.86 ^b^	197.66 ^f^	142.6 ± 0.2 ^k^
Cyclohexane	353.9 ± 0.2 ^a^	33.1 ± 0.4 ^a^	29.97 ^b^	156.12 ^d^	105.3 ± 2.0 ^l^
Benzene	353.3 ± 0.1 ^a^	33.9 ± 0.1 ^a^	30.72 ^b^	136.24 ^g^	82.44 ^j^
Acetonitrile	354.8 ± 0.4 ^a^	33.4 ^b^	29.75 ^b^	91.69 ^h^	-
Ethyl acetate	350.2 ± 0.2 ^a^	35.0 ± 2.0 ^a^	31.94 ^b^	169.30 ^i^	113.64 ^m^
Tetrachloromethane	349.8 ± 0.3 ^a^	32.0 ± 2.0 ^a^	29.82 ^b^	131.40 ^d^	-

^a^ Reference [24]. ^b^ Reference [27]. ^c^ Reference [28]. ^d^ Reference [29]. ^e^ Reference [30]. ^f^ Reference [31]. ^g^ Reference [32]. ^h^ Reference [33]. ^i^ Reference [34]. ^j^ Reference [35]. ^k^ Reference [36]. ^l^ Reference [37]. ^m^ Reference [38].

**Table 7:** Compositions *x*_A_, boiling points *T*_Az_, and excess enthalpies *H*^E^ at *T* = 298.15 K of the binary azeotropes studied. The name of component A in each mixture is underlined.

Azeotrope	*x*_A_^a^	*T*_Az_/K	*H*^E^/(kJ∙mol^−1^)
Benzene + methanol	0.6190	331.56 ^b^	0.53 ^c^
Benzene + ethanol	0.4550	341.25 ^b^	0.83 ^c^
Benzene + 1-propanol	0.7858	350.20 ^b^	0.85 ^d^
Cyclohexane + ethanol	0.4708	337.95 ^b^	0.64 ^e^
Cyclohexane + 1-propanol	0.7858	347.68 ^b^	0.56 ^d^
Cyclohexane +1-butanol	0.9295	352.68 ^b^	0.38 ^f^
Acetonitrile + benzene	0.5200	346.05 ^h^	0.44 ^i^
Acetonitrile + ethyl acetate	0.6499	-	-
Acetonitrile + tetrachloromethane	0.5811	338.25 ^b^	0.79 ^d^
*n*-Hexane + ethanol	0.6730	331.65 ^b^	-
*n*-Hexane + 1-propanol	0.9288	338.95 ^j^	-
*n*-Hexane + 1-butanol	0.9593	341.35 ^b^	0.29 ^g^

^a^ Reference [14]. ^b^ Reference [39]. ^c^ Reference [40]. ^d^ Reference [41]. ^e^ Reference [42]. ^f^ Reference [43]. ^g^ Reference [44]. ^h^ Reference [45]. ^i^ Reference [46]. ^j^ Reference [47].

## Data Availability

Data are included within the article.
